# Patterns in Child Health Outcomes Before and After the COVID-19 Outbreak in India

**DOI:** 10.1001/jamanetworkopen.2023.17055

**Published:** 2023-06-05

**Authors:** Soohyeon Ko, Rockli Kim, S. V. Subramanian

**Affiliations:** 1 Department of Public Health Sciences, Graduate School of Korea University, Seoul, Republic of Korea; 2 Interdisciplinary Program in Precision Public Health, Korea University, Seoul, Republic of Korea; 3 Division of Health Policy and Management, College of Health Science, Korea University, Seoul, Republic of Korea; 4 Harvard Center for Population and Development Studies, Cambridge, Massachusetts; 5 Department of Social and Behavioral Sciences, Harvard T.H. Chan School of Public Health, Boston, Massachusetts

## Abstract

This cross-sectional study examines prepandemic-to-postpandemic changes in mortality, nutrition and feeding practices, anthropometry, vaccination, and other measures in a sample of children from the Indian National Family Health Survey.

## Introduction

The COVID-19 pandemic and subsequent national lockdowns in many countries disrupted access to basic services.^1^ Several welfare programs were put in place, even in resource-limited settings, to mitigate the socioeconomic and health consequences.^2^ To assess the overall implication of COVID-19 for population health, data ideally should be collected immediately before and after the outbreak. We used the 2019 to 2021 National Family Health Survey (NFHS)^3^ in India, a country with the second-highest number of COVID-19 cases and the third-highest death tolls in the world as of January 2023,^4^ to examine the systematic differences in various child health outcomes before vs after the outbreak.

## Methods

The cross-sectional data of the 2019 to 2021 NFHS provided a unique opportunity to perform an empirical assessment, as the data were collected both before and after March 2020, when the national lockdown was declared in 13 of the 36 states or Union Territories in India, facilitating a natural comparison in health outcomes. For this cross-sectional study, data collected from June 17, 2019, to February 29, 2020, were defined as *before COVID-19* and those from March 1, 2020, to May 20, 2021, were defined as *after COVID-19*. Further details on the survey design are available elsewhere.^3^ The Harvard Longwood Campus Institutional Review Board deemed this study exempt from ethics review because it was a secondary use of anonymized information. This study followed the STROBE reporting guideline.

Child health outcomes with a short reference period and deemed most likely to be affected by disruptions in health services were selected (Table 1). Twenty-six indicators related to pregnancy and child health and health care, feeding and nutrition, anthropometric failures, and vaccination were included. Absolute differences (in percentage points) were calculated by comparing the prevalence of outcomes before vs after the outbreak (eg, prevalence [stunting] after COVID-19 − prevalence [stunting] before COVID-19).

**Table 1.** Descriptive Statistics of the Analytic Sample From 2019 to 2021 Indian National Family Health Survey^a^

Indicator	Total No.^b^	Age, mean (SD), mo^c^	Male, No. (%)^c^	Female, No. (%)^c^
Pregnancy-related				
Antenatal care: ≥4 visits	93 848	25.58 (16.99)	51 022 (54.34)	42 826 (45.66)
Antenatal care: ≥8 visits	93 848	25.58 (16.99)	51 022 (54.34)	42 826 (45.66)
Skilled birth attendance	125 812	29.95 (17.55)	65 574 (52.14)	60 238 (47.86)
In-facility delivery	125 812	29.95 (17.55)	65 574 (52.14)	60 238 (47.86)
Health and health care				
Neonatal mortality within 28 d of birth	125 812	29.95 (17.55)	65 574 (52.14)	60 238 (47.86)
Low birth weight	110 161	29.67 (17.49)	57 327 (52.11)	52 834 (47.89)
Diarrhea	120 378	29.92 (17.55)	62 575 (52.02)	57 803 (47.98)
Diarrhea treated with ORS	7082	23.48 (16.31)	3769 (53.59)	3313 (46.41)
Diarrhea treated with zinc	6891	23.49 (16.33)	3676 (53.74)	3215 (46.26)
Diarrhea treated at health facility or by health care practitioner	7093	23.49 (16.31)	3777 (53.62)	3316 (46.38)
ARI	120 419	29.92 (17.55)	62 592 (52.02)	57 827 (47.98)
Fever or symptoms of ARI treated at health facility or by health care practitioner	10 914	27.18 (16.60)	5942 (53.87)	4972 (46.13)
Feeding and nutrition				
Receiving solid or semisolid food	5751	7.40 (0.96)	2978 (50.84)	2773 (49.16)
Adequate diet for children who had breastfeeding	28 117	13.74 (5.04)	14 810 (52.37)	13 307 (47.63)
Adequate diet for children who had no breastfeeding	4273	16.53 (4.64)	2171 (49.88)	2102 (50.12)
Anemia	97 032	33.30 (15.54)	50 637 (52.27)	46 395 (47.73)
Integrated Child Development Services	41 390	28.75 (17.02)	21 614 (52.18)	19 776 (47.82)
Anthropometric failure				
Wasting	108 458	30.12 (17.32)	56 223 (51.89)	52 235 (48.11)
Underweight	112 661	29.75 (17.45)	58 568 (52.06)	54 093 (47.94)
Stunting	110 507	30.03 (17.39)	57 391 (52.00)	53 116 (48.00)
Overweight	108 458	30.12 (17.32)	56 223 (51.89)	52 235 (48.11)
Vaccination				
BCG vaccine	71 597	17.59 (10.44)	37 140 (51.73)	34 457 (48.27)
First dose of DPT vaccine	27 566	6.49 (3.88)	14 229 (51.21)	13 337 (48.79)
Third dose of DPT vaccine	27 566	6.49 (3.88)	14 229 (51.21)	13 337 (48.79)
First dose of polio vaccine	27 591	6.49 (3.88)	14 240 (51.20)	13 351 (48.80)
Third dose of polio vaccine	27 591	6.49 (3.88)	14 240 (51.20)	13 351 (48.80)

Abbreviations: ARI, acute respiratory infection; BCG, bacille Calmette-Guérin; DPT, diphtheria, pertussis, tetanus; ORS, oral rehydration salts.

^a^ This analytic sample was restricted to 13 states or Union Territories with survey data that were collected immediately before and after the COVID-19 outbreak: Punjab, Uttarakhand, Haryana, National Capital Territory of Delhi, Rajasthan, Uttar Pradesh, Arunachal Pradesh, Jharkhand, Odisha, Chhattisgarh, Madhya Pradesh, Tamil Nadu, and Puducherry.

^b^ Excluding individuals with missing data on each outcome variable. Thus, the sample size for each outcome varied.

^c^ Descriptive statistics were estimated using survey weight.

Statistical significance was determined using logistic regression models adjusted for child age, sex, and state fixed effects. To account for the multistage, stratified cluster-sampling design, survey weights were applied to all statistical analyses. Two-sided *P* = .05 indicated statistical significance. Analyses were performed between October 2022 and January 2023, using Stata 17 (StataCorp LLC).

## Results

The sample size for the most complete outcome was 125 812 (65 574 boys [52.1%], 60 238 girls [47.9%]; mean [SD] age, 30.0 [17.6] months) (Table 1). Compared with before-COVID-19 data, after-COVID-19 data showed small but significant deterioration in neonatal mortality (0.49 percentage points), feeding and nutrition (eg, 4.22 percentage points reduction in solid or semisolid food intake), and anthropometric failures (eg, 1.87 percentage points increase in underweight) (Table 2). The most substantial difference was found in vaccination indicators, with 7.74 percentage points and 6.51 percentage points reduction in first dose of DPT (diphtheria, pertussis, tetanus) and polio, respectively. Other indicators, including many related to health services, either remained constant or marginally improved during the outbreak.

**Table 2.** Comparison of Selected Child Health Indicators Before and After COVID-19 Outbreak From 2019 to 2021 Indian National Family Health Survey^a^

Indicator	COVID-19 outbreak, No. (weighted %)^b^		Absolute difference, percentage point	Direction of change	*P* value^c^
	Before	After			
Pregnancy-related					
Antenatal care: ≥4 visits^d^	51 626 (56.56)	857 (54.74)	−1.82	Deterioration	.28
Antenatal care: ≥8 visits^d^	13 559 (16.36)	173 (12.60)	−3.76	Deterioration	.95
Skilled birth attendance^e^	80 013 (71.95)	10 082 (72.35)	0.40	Improvement	.005
In-facility delivery^e^	97 662 (88.21)	12 500 (90.07)	1.86	Improvement	.11
Health and health care					
Neonatal mortality within 28 d of birth^e^^,^^f^	3003 (2.72)	425 (3.21)	0.49	Deterioration	.03
Low birth weight^e^^,^^f^	17 901 (18.53)	2342 (18.62)	0.09	Deterioration	.81
Diarrhea^g^	2940 (7.82)	4153 (4.94)	−2.88	Improvement	<.001
Diarrhea treated with ORS^g^	1687 (57.49)	2519 (58.01)	0.52	Improvement	.09
Diarrhea treated with zinc^g^	812 (27.73)	1438 (34.26)	6.53	Improvement	<.001
Diarrhea treated at health facility or by health care practitioner^g^	2030 (68.99)	2981 (72.91)	3.92	Improvement	<.001
ARI^g^	1720 (4.52)	1652 (2.08)	−2.44	Improvement	<.001
Fever or symptoms of ARI treated at health facility or by health care practitioner^g^	3793 (74.14)	4247 (74.77)	0.63	Improvement	.05
Feeding and nutrition					
Receiving solid or semisolid food^g^	774 (44.36)	1638 (40.14)	−4.22	Deterioration	<.001
Adequate diet for children who had breastfeeding^g^	952 (10.66)	1958 (8.41)	−2.25	Deterioration	<.001
Adequate diet for children who had no breastfeeding^g^	151 (13.21)	323 (10.93)	−2.28	Deterioration	.02
Anemia^g^	21 590 (71.33)	44 188 (65.86)	−5.47	Improvement	<.001
Integrated Child Development Services^g^	16 735 (67.62)	11 887 (69.60)	1.98	Improvement	.87
Anthropometric failure					
Wasting^g^	5666 (16.66)	12 769 (17.00)	0.34	Deterioration	.22
Underweight^g^	9887 (28.25)	23 315 (30.12)	1.87	Deterioration	<.001
Stunting^g^	12 052 (34.93)	26 304 (34.37)	−0.56	Improvement	.01
Overweight^g^	1371 (3.75)	2566 (2.96)	−0.79	Improvement	<.001
Vaccination					
BCG vaccine^e^	54 887 (94.45)	12 487 (92.05)	−2.4	Deterioration	<.001
First dose of DPT vaccine^e^	11 304 (80.17)	9845 (72.43)	−7.74	Deterioration	.57
Third dose of DPT vaccine^e^	8171 (57.54)	6134 (45.20)	−12.34	Deterioration	.002
First dose of polio vaccine^e^	11 090 (78.99)	9822 (72.48)	−6.51	Deterioration	.03
Third dose of polio vaccine^e^	7593 (53.60)	5807 (42.73)	−10.87	Deterioration	<.001

Abbreviations: ARI, acute respiratory infection; BCG, bacille Calmette-Guérin; DPT, diphtheria, pertussis, tetanus; ORS, oral rehydration salts.

^a^ This comparison was restricted to 13 states or Union Territories with survey data that were collected immediately before and after the COVID-19 outbreak: Punjab, Uttarakhand, Haryana, National Capital Territory of Delhi, Rajasthan, Uttar Pradesh, Arunachal Pradesh, Jharkhand, Odisha, Chhattisgarh, Madhya Pradesh, Tamil Nadu, and Puducherry.

^b^ Weighted prevalence was estimated using survey weight.

^c^ *P* values were based on state fixed-effect logistic regression models adjusted for child age and sex.

^d^ Before and after COVID-19 periods were defined as pregnancy occurring before or after COVID-19 outbreak, respectively.

^e^ Before and after COVID-19 periods were defined as births occurring before or after COVID-19 outbreak, respectively.

^f^ Age was not adjusted for these outcomes.

^g^ Before and after COVID-19 periods were defined as National Family Health Survey interview occurring before or after COVID-19 outbreak, respectively.

## Discussion

Mixed results from this analysis suggested that adverse consequences of COVID-19 and national lockdown were countered, to some extent, by emergency relief programs. For example, the Indian government launched Pradhan Mantri Garib Kalyan Ann Yojana in 2020 to distribute 5 kg of food grains and 1 kg of pulses per month to approximately 800 million individuals (approximately two-thirds of India’s population).^5^ This initiative may explain the relatively constant or minimally worsened patterns in child nutrition status before and after the outbreak. It also underscored the need to sustain relief programs in nonpandemic times to promote children’s health. Improvements in child health outcomes, such as diarrhea and acute respiratory infection rates, may be attributed to the wider promotion of interpersonal hygiene during the pandemic.^6^

Study limitations included the cross-sectional design, which prohibited any causal inferences from being drawn, and the inability to distinguish COVID-19’s implications from those of longer-term exposures to harmful conditions. Nevertheless, the results showed that nationally representative surveys, even with COVID-19-related disruptions in data collection, can aid in understanding the pandemic’s outcome.

## Notes

Supplement.
Data Sharing Statement
