# Supplementary material for: Patterns in Child Health Outcomes Before and After the COVID-19 Outbreak in India
Source: JAMA Netw Open. 2023 Jun 5;6(6):e2317055. doi: 10.1001/jamanetworkopen.2023.17055 (PMC10242422; doi:10.1001/jamanetworkopen.2023.17055)
Supplement: Supplement. — Data Sharing Statement [file jamanetwopen-e2317055-s001.pdf]

Data Sharing Statement

Ko. Patterns in Child Health Outcomes Before and After the COVID-19 Outbreak in India.
JAMA Netw Open. Published June 05, 2023. doi:10.1001/jamanetworkopen.2023.17055

Data
Data available: No

Additional Information
Explanation for why data not available: Data used for this analysis can be downloaded from: https://dhsprogram.com/
